# Effective suckling in relation to naked maternal-infant body contact in the first hour of life: an observation study

**DOI:** 10.1186/1471-2393-14-20

**Published:** 2014-01-14

**Authors:** Ruth M Cantrill, Debra K Creedy, Marie Cooke, Fiona Dykes

**Affiliations:** 1 Metro South Hospital and Health Service, Queensland Health, PO Box 7254, Redland Bay, Queensland 4165, Australia; 2 Griffith Health Institute Griffith University, Nathan, Queensland 4111, Australia; 3 Maternal and Infant Nutrition and Nurture Unit (MIANN), University of Central Lancashire, Lancashire, UK

**Keywords:** Newborn feeding behaviour, Breastfeeding initiation, Skin-to-skin contact, Observation, Best practice guidelines, Baby Friendly Hospital Initiative

## Abstract

**Background:**

Best practice guidelines to promote breastfeeding suggest that (i) mothers hold their babies in naked body contact immediately after birth, (ii) babies remain undisturbed for at least one hour and (iii) breastfeeding assistance be offered during this period. Few studies have closely observed the implementation of these guidelines in practice. We sought to evaluate these practices on suckling achievement within the first hour after birth.

**Methods:**

Observations of seventy-eight mother-baby dyads recorded newborn feeding behaviours, the help received by mothers and birthing room practices each minute, for sixty minutes.

**Results:**

Duration of naked body contact between mothers and their newborn babies varied widely from 1 to 60 minutes, as did commencement of suckling (range = 10 to 60 minutes). Naked maternal-infant body contact immediately after birth, uninterrupted for at least thirty minutes did not predict effective suckling within the first hour of birth. Newborns were four times more likely to sustain deep rhythmical suckling when their chin made contact with their mother’s breast as they approached the nipple (OR 3.8; CI 1.03 - 14) and if their mothers had given birth previously (OR 6.7; CI 1.35 - 33). Infants who had any naso-oropharyngeal suctioning administered at birth were six times less likely to suckle effectively (OR .176; CI .04 - .9).

**Conclusion:**

Effective suckling within the first hour of life was associated with a collection of practices including infants positioned so their chin can instinctively nudge the underside of their mother’s breast as they approach to grasp the nipple and attach to suckle. The best type of assistance provided in the birthing room that enables newborns to sustain an effective latch was paying attention to newborn feeding behaviours and not administering naso-oropharyngeal suction routinely.

## Background

The Baby Friendly Hospital Initiative (BFHI) recommends that newborn babies be placed in skin-to-skin contact with their mothers immediately following birth for at least one hour, and mothers helped to initiate breast feeding within the first half-hour following birth [1,2]. ‘Ten Steps to Successful Breastfeeding’ were adopted as the minimum standard for hospitals worldwide to gain accreditation as “Baby Friendly” [3]. Of these ten best practice standards, ‘Step 4’ (management of the first breastfeed) has been criticised as ambiguous and most often neglected in practice [4-6].

The term ‘skin-to-skin contact’ is defined as ‘naked newborn placed on his/her mother’s bare chest’ and is widely used in practice. However some mothers and midwives may misunderstand the term and believe that placing babies cheek to cheek with the mother or resting the baby’s face on their mother’s breast might be ‘skin-to-skin’. We argue that the term ‘naked body contact’ more clearly portrays the concept of naked baby on mother’s bare chest. In this paper, the term ‘skin-to-skin contact’ will be used when reference is made to existing research studies and ‘naked body contact’ will be used for presenting the findings of the present study.

The benefits of skin-to-skin contact between mother and newborn during the first hours after birth are well known [7-15]. Skin-to-skin contact between a mother and her newborn enhances both newborn feeding ability and behaviours conducive to suckling ability [16-19]. Righard and Alade observed that newborn babies are able to attach to the breast independently and suckle effectively if allowed continuous uninterrupted skin-to-skin contact with their mothers for one hour following birth [20,21]. Widstrom and Thingstrom-Paulsson observed that the position of the newborn’s chin provides a reference point to help them gape widely, grasp the nipple and take in adequate breast tissue to commence suckling while held by their mothers in skin-to-skin contact from the moment of birth [18].

The first 2 hours after birth is a sensitive period in which mothers are particularly responsive to their newborns [22,23]. Recent research suggests skin-to-skin contact between mother and newborn can improve breastfeeding initiation rates and duration of breastfeeding [24,25]. Furthermore, when suckling at the breast is achieved within the first hours after birth, infant mortality and morbidity are reduced [26].

BFHI accreditation standards recommend that mothers receive help to breastfeed within 30 minutes of birth and emphasize ‘*correct’* positioning and ‘*correct’* attachment [2,27]. However, guidance and definitions of how to assist mothers in the birthing room are not clear. Although a ‘hands off’ approach to help mothers with positioning and attachment has been suggested as effective in the postnatal ward [28-31], midwives are reported to use a ‘hands on’ approach with the first breastfeed [32] in the birthing suite. There is growing evidence to suggest babies have an innate ability to find the nipple and commence breastfeeding [18,20,33]. Widstrom et al. observed nine behavioural phases when newborns were in skin-to-skin contact with their mothers for the first hour following birth, birth cry, relaxation, awakening, activity, crawling, resting, familiarization, suckling and sleeping [15]. These findings suggest that a hands-off approach may also be useful for breastfeeding initiation in the birthing suite.

To our knowledge, no published studies have explored relationships between time newborns are in skin-to-skin contact and effectiveness of suckling. Furthermore, there has been very little research on the extent of help mothers receive in the birthing room. Descriptions of best practice for breastfeeding commencement could inform birthing room protocols. The aim of this study was to identify predictors of newborn suckling behaviour within the first of hour after birth. It was hypothesised that naked newborn infants placed immediately on their mothers’ bare chest after birth and left undisturbed for at least 30 minutes would commence breastfeeding and suckle effectively sooner than infants not receiving this level of contact.

## Method

### Sample

Pregnant women over 18 years of age and intending to breastfeed were recruited from the antenatal clinic of the participating hospital. Participants were at least 36 weeks gestation, able to communicate in English, and with no major prenatal complications or underlying medical problems likely to impact on their ability to hold their naked baby immediately after or within 30 minutes of birth. One hundred and three women enrolled in the study. Participating women returned a signed consent form and completed demographic information plus a breastfeeding intention/history questionnaire. In terms of the recommended ratio of variables to subjects [34,35] the 78 cases used in the analysis was considered adequate, given that only four variables were employed as predictors.

### Setting

The study was conducted within the Women and Birthing Services at a public metropolitan hospital in Queensland, Australia. The site was preparing for BFHI accreditation. Approval by the Human Research Ethics Committees for Griffith University and Metro South Health Service District was obtained.

### Measures

Personal information such as age, income and education and obstetric data in relation to wellbeing, previous pregnancies and breastfeeding history was sought.

### Birthing room observation tool

The birthing room observation tool was devised using a time line grid with items listed down one side. The list of items was developed in consultation with midwifery colleagues and drew on standard maternity procedures, perinatal data collection forms [36], and a critical review of the research literature concerning newborn feeding behaviour, how mothers respond to their infants [37,38], and recommendations for support and assistance to mothers [2,39-45]. An expert panel of seven professionals including clinical nurse midwives, International Board Certified Lactation Consultants (IBCLC) and researchers reviewed the list of items.

The observation tool was based on four overarching sets of variables related to: 1) Birthing events and placement of babies immediately following birth; 2) Newborn feeding behaviours; 3) Maternal behaviour to initiate breastfeeding; and 4) Assistance mothers received to position and attach their baby to the breast.

Birthing events and placement of babies immediately following birth consisted of 65 items. There were 5 sub-categories. i) Birth: items recorded information such as time of birth and immediate observations associated with the birth for example, baby weak/vigorous cry. ii) Baby placed: determined what happened to babies from the first moment of birth and at any time throughout the hour, for example, if babies were put on their mother’s abdomen, resuscitation cot or elsewhere. iii) Maternal response to baby: recorded how mothers responded to their infants, and the point in time mothers held their babies, for example, mother picks newborn up, holds baby on bare skin. iv) Baby held: ascertained by whom babies were held, at what point in time and whether wrapped, covered or clothed, for example, by mother, by father, loosely wrapped, nappy on. v) Resuscitation: verified the point in time any resuscitation event occurred such as mouth/nose suction, nasal oxygen [46]. From these observations, the point in time and the length of time babies were held naked on their mothers’ bare chest or abdomen could be calculated and any barriers or interruptions to naked body contact between mothers and their newborns could be identified.

2) Newborn feeding behaviours had 32 items. This was a list of expected, known, and defined feeding behaviours [17,20,37,47,48] leading up to and including sustained suckling e.g. lifts head up, nudges breast with chin, suckles at breast.

3) Maternal behaviour to initiate breastfeeding had 21 items. This section clarified actions mothers took to position themselves or their baby to offer their babies a breastfeed at any point in time, e.g. mother positions herself, positions baby close etc.

4) Assistance mothers received to position and attach their baby to the breast had 19 items such as breastfeeding assistance offered, baby held to breast for mother, and readiness to breastfeed discussed [2]. This described any assistance mothers received with positioning and attachment of their newborn baby to the breast and at what point in time.

The tool was pilot tested on two births before commencing data collection. Three new observations were added and several repetitious items deleted in order to refine the tool.

### Procedure

Midwives who worked regularly in the birthing suite (n = 23 out of a possible 40 permanent midwifery staff) agreed to the researcher being present and recording observations of consenting mothers in their care. Midwives were requested to not make changes to their normal practice, and behave as if the researcher was not present. Staff at the Unit informed the researcher of a participating woman’s admission. The researcher then arrived at the birthing room, confirmed the consent of the woman and her partner and gained permission to be present from the person managing the birth.

Equipment for observations included the observation record sheet on a clip board, a stop-watch, a tape recorder, ear phones, and a pen. When birth was imminent, the researcher entered the room to observe. At the moment of birth the researcher pressed the stop-watch, recorded the time of birth and pressed the play button on the tape recorder [49]. A pre-recorded tape of a soft ‘beep’ every minute reminded the researcher so events could be recorded accurately as they occurred by ticking the space next to the listed item at the specific time. The first author (RC) observed all births.

Observations were unobtrusive with no interaction with mothers, family or staff [49,50]. A good view of the baby exhibiting feeding behaviours was obtained by standing at the back of the head of the bed or to the side while coming in a little closer to view actions such as mouthing, licking, latching and suckling. At the end of the hour, the researcher thanked the parents and staff before leaving the room. Data were collected between January and July 2004.

### Data preparation

Data were prepared for analysis in two steps. The first step was data cleaning and data entry in Microsoft Excel prior to importation to the Statistical Package for the Social Sciences (SPSS) [51]. The second step was to identify and choose variables of interest from the lists of observations. Three processes established variables and definitions.

i) Continuous variables measured uninterrupted naked body contact time between mother and newborn or time mothers and babies were separated as well as time between, before or after specific events. For example, time infants suckled effectively and longest time mothers’ and newborns’ spent in naked body contact were calculated as continuous variables that could be categorized.

ii) Categorical variables were developed from the continuous data as well as from raw data collected such as ‘mode of birth’. Length of time or ‘duration of naked body contact’ was calculated from the first moment baby was placed naked on his/her mother’s bare chest to the time baby was removed. Categorical dichotomous variables were created to measure whether or not naked body contact was interrupted, and the mother, infant or staff performed various behaviours. Four categories of ‘continuous uninterrupted’ and ‘interrupted naked body contact’ were ‘0 = naked body contact immediately after birth and continued undisturbed for at least 30 to 60 minutes’, ‘1 = naked body contact interrupted within 1–5 minutes then commenced or continued for at least 30 minutes’, ‘2 = naked body contact commenced after 5 minutes and interrupted before 30 minutes’, ‘3 = no naked body contact at all or none after the first minute’.

iii) The third process used to determine variables for analysis involved an analysis of items observed in the birthing room using Principal Components Analysis (PCA) [35,52,53]. This identified components constructed from items that correlated with each other, or clustered together. Hence the dependent variable (DV) ‘effective suckling’ meant newborns who took an asymmetrical latch and sustained deep rhythmical suckles for at least four minutes. Babies for whom this was true were given a score of ‘1 = yes, effective suckling for at least four minutes during the hour’ and the remainder a score of ‘0 = no, did not suckle effectively at any time during the 60 minutes’. Other independent variables (IV) such as ‘mother initiates breastfeeding’ and ‘assistance to attach’ are described under definitions.

### Definitions of independent variables

#### ***Duration of naked body contact***

Naked body contact (also known as skin-to-skin contact) means the baby is naked (with the exception of a nappy) and is lying face down on his/her mothers’ bare chest. As described above, ‘continuous uninterrupted naked body contact’ means no interruption of maternal-infant naked body contact from the moment of birth to at least 30 minutes. ‘Interrupted naked body contact’ refers to maternal-infant naked body commenced between 2–5 minutes after birth or interrupted within the first 5 minutes of birth and resumed for at least 30 minutes. ‘Naked body contact later’ means maternal-infant naked body contact commenced later than 5 minutes after birth. Cases where babies were placed with their mother on top of clothing or bed linen or wrapped were not considered naked body contact until the moment they were actually placed bare on the mother’s bare chest.

#### ***Feeding behaviour and suckling***

‘Chin contact breast’ means the baby’s chin nudged mother’s breast as he/she approached to suckle either independently or with assistance. The single item ‘suckled at the breast for the first time’ could be verified without doubt by mother and midwife and significant others involved and were defined as ‘commencement of suckling’ or suckling achievement. ‘Effective suckling’ was defined as deep rhythmical suckles sustained for at least four minutes.

#### ***Maternal behaviours***

‘Mother initiates breastfeeding’ was where mothers acted independently to position themselves or their babies to offer their babies a breastfeed. ‘Positioning and entice to attach’ was constructed from any one of four items, ‘turns baby to face herself’, ‘positions baby close’, ‘expresses colostrum for baby or ‘holds nipple to baby’s mouth’.

#### ***Assistance received***

The term ‘assisted to attach’ was defined by one or more actions occurring (baby held to the breast for the mother, baby’s neck held by ‘tong’ grip or mother’s breast shaped to help baby attach to the breast). Mothers who received hands on assistance to attach baby to the breast regardless of other help received were deemed to be in the category of ‘assisted to attach’. The IV ‘head held and assist to attach’ meant the back of baby’s head was held when ‘assisted to attach’.

Definitions for other categorical dichotomous variables were ‘suction’ defined as any nasal, oral, pharyngeal or gastric suction administered to babies. ‘Analgesia’ refers to either Pethidine injection, epidural or spinal anaesthesia. Parity is the number of live born children a woman has given birth to. Primapara are first time mothers and multipara are women who have given birth to two or more live born children.

#### ***Statistical analysis***

Data were analysed using International Business Machines (IBM) SPSS version 21 [51]. An alpha level of .05 was used for all statistical tests and a 95% confidence interval was used for all estimated values. Chi-square analyses were used to examine the association between whether 1) suckling commenced and 2) effective suckling occurred and each proposed predictor. Independent variables with a p-value ≤ 0.05 were retained for logistic regression. Direct logistic regression (where all predictors are entered into the equation simultaneously and there are no specific hypotheses about the order or importance of predictor variables) was performed to identify predictors of effective suckling within the first hour of life [35,54,55].

Proposed predictor variables were chosen as described above based on clinical observation and experience [40,56], the research literature [15,17,20,31,57-59] and BFHI policy guidelines [27] and PCA. Ideally predictor variables are strongly related to the DV but not to each other [34,35,55]. Independent variables (IVs) were tested for correlation with effective suckling and also for muticollinearity [35,55]. No muticollinearity issues between predictor variables were noted [34,35,55]. Chosen predictors were entered into the direct logistic regression model. Categories of naked body contact duration were collapsed to three categories of ‘0 = continuous uninterrupted naked body contact’ (reference category coded ‘0’) [35] ‘1 = interrupted naked body contact’ and ‘2 = naked body contact later or not at all’. Categories were based on BFHI recommendations that newborns be placed naked on their mothers’ bare chest immediately or within five minutes following birth and remain undisturbed for at least 1 hour. The ‘goodness of fit’ of the logistic regression model was assessed by the Hosmer and Lemeshow Test where a non-significant result indicates support for the model.

## Results

**Figure 1:**
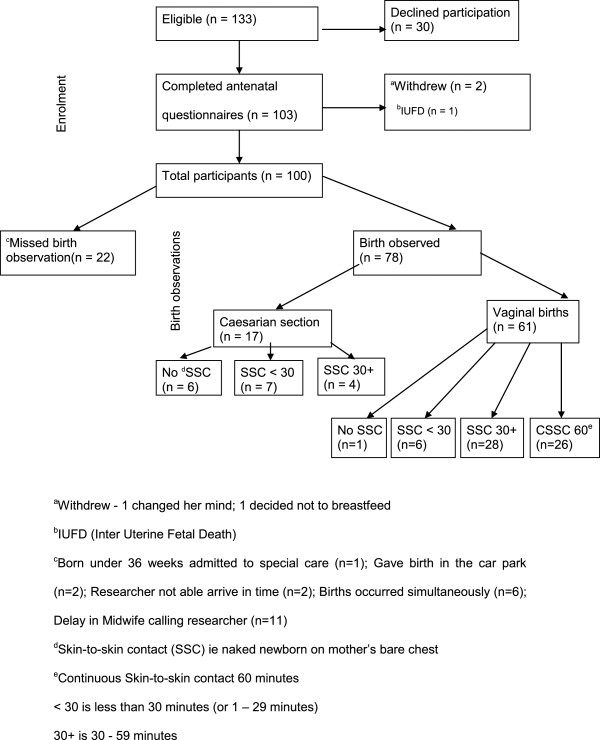
Response rate, births observed.

Of recruited mothers (n = 103), 78 births were observed (Figure 1). Births were missed (n = 22) if the attending midwife delayed or did not notify the researcher of an impending birth (n = 11), if infant born less than 36 weeks gestation, if the researcher was unable to attend (n = 2), if births occurred simultaneously (n = 6) or if a birth occurred too quickly (n = 2) (precipitate birth) e.g. in the car park. Demographic characteristics of the sample are shown in Table 1[36,60,61]. The sample was similar to the State birthing population for age and marital status. Labor and birthing outcomes compared with the state of Queensland (Table 2) [36].

**Table 1:** Demographic characteristics of sample

**Demographic characteristics**	**Births observed n (%)**	**State or national population data (%)**
**Age**^**a**^		
18-19 years	3 (3.9)	(5.6)^b^
20-24	12 (15.4)	(17.5)
25-29	26 (33.3)	(27.7)
30-34	27 (34.6)	(31.3)
35 and over	10 (13)	(17.9)
Marital status^a^		
Married/defacto	72 (91.3)	(87.6)
Single/separated/divorced	5 (6.6)	(12.3)
Non-response	1 (1.3)	-
**Occupational category**^**c**^		
Home duties	18 (27.7)	-
Clerical/ & administrative	18 (27.7)	(25)
Community & personal service	8 (10.3)	(14)
Manager/	7 (10.8)	(9)
Technicians & trades	5 (7.7)	(5)
Sales	4 (6.2)	(15)
Professional/	3 (4.6)	(20)
Student	2 (3.1)	-
Non-response	13 (16.7)	(1)
**Income**^**c**^		
No Income	-	(6)
Up to $22,500	6(7.7)	(31)
$22,501 - 55,500	37 (47.4)	(43)
$55,501 or more	29 (37.2)	(13)
Non-response	6(7.7)	(7)
**Education**^**d**^		
Secondary education	41 (52.6)	(69)
Tertiary study	18 (23.1)	(28.6)
Higher degree	15 (20.5)	(36.7)
Non-response	3 (3.8)	-

^a^Queensland Health (2006), Health Statistics Center, Perinatal Statistics 2005.

^b^Queensland Perinatal Data category is 15 – 19 years.

^c^Australian Bureau of Statistics (ABS) (2007), 2006 Census Community Profile (based on women in Queensland aged 18 – 54).

^d^Queensland Government. (2006). Department of Education and the Arts.

**Table 2:** Labor and birth

	**Births observed**	**Queensland perinatal data (2005)**
	**n (%)**	**(%)**
**Onset of labor**		
Spontaneous	43 (55.1)	(57.2)
Induced	25 (32.1)	(23.4)
No labour (c/s)	10 (12.8)	(19.5)
**Adverse birth event**		
Prolonged 2nd stage &/or failure to progress	9 (11.5)	(12.4)
Foetal distress &/or meconium liquor	12 (15.4)	(20.2)
Labour <4 hours (precipitate birth)	10 (14.7)	(23.5)
**Method of Analgesia during labor**^**a**^		
At least one method	55 (70.5)	(63.6)
Nitrous oxide	52 (66.7)	(62.4)
Pethidine (narcotic IM/IV)^b^	22 (28.2)	(35.3)
Epidural	24 (30.8)	(33.9)
Spinal anaesthesia/other	11 (14.1)	(10.1)
**Vaginal birth**		
Spontaneous vertex	52 (66.7)	(59.5)
Forceps/vacuum extraction	9 (11.5)	(7.9)
**Caesarean section**		
Elective caesarean section	10 (12.8)	(19.5)
Emergency caesarean section	7 (9)	(13)
**Episiotomy**	11 (14.1)	(13.5)
Baby **Apgar score** @ 1 minute		
Score of 6 – 7^c^	8 (6.5)	(14.8)
Score of 8 - 10	70 (89.7)	(83.2)
**Baby Apgar score** @ 5 minutes		
Score of 8 - 10	78 (100)	(97.4)
**Birth weight by gestation**		
2000 - 2449 grams		
36 weeks gestation (32–36)^d^	1 (1.3)	(1.8)
37–41 weeks gestation	4 (5.1)	(1.5)
2500 grams - 3999		
37–41 weeks gestation	58 (74.4)	(78.3)
4000 grams plus		
37 plus weeks gestation	15 (19.2)	(15.8)
**Sex**		
Male	40 (51.3)	(51.5)
Female	38 (48.7)	(48.5)

^a^Multiple methods of analgesia/anaesthesia included.

^b^Intramuscular (IM) or Intravenous (IV).

^c^Perinatal Data range is 4–6 for State of Queensland.

^d^Perinatal Data range is 32–36 weeks gestation for State of Queensland.

### Suckling achievement

Fifty-three of the babies (68%) began suckling within 60 minutes following birth. The average time to begin suckling was 38 minutes (SD 14.5; median 37; IQR 24.5 - 51). Thirty babies (38.5%) ‘breastfed well’ with deep rhythmical suckles sustained for at least four minutes. Twenty- three (29.5%) achieved ‘a few suckles’ or suckled for one to three minutes, fourteen (61%) of these made ‘repeated attempts’ before attaching to suckle. Of the twenty-five (32%) babies who did not suckle, twelve (48%) were unable to latch despite ‘attempts to grasp the nipple’ while nine (36%) only mouthed the nipple or ‘licked and nuzzled’ and four (16%) made no attempt as if ‘not interested in feeding’.

PCA identified four components that explained a total of 46% of the variance of newborn feeding behaviours. ‘Effective suckling’ contributed 18.5% of the variance and consisted of three items ‘grasp nipple for the first time’, ‘suckles at breast for the first time’ and ‘sustained suckle (deep rhythmical suckles)’. ‘Hand to mouth co-ordination’ comprised eight pre-feeding behaviours that explained 12.6% of the variation. Five items related to newborn ‘head movement’ contributing 7.4% were ‘turns head’, ‘lifts head up’, ‘nudges breast with chin’, ‘wide gape’ and ‘attempt to grasp nipple’. ‘Attempt to attach’ contributed 7.3% with items ‘attempt to grasp nipple’, ‘nudges breast with chin’, ‘wide gape’ and ‘attempt to turn head’.

### Duration of undisturbed naked body contact

Many inconsistencies in practice of naked body contact between mother and newborn during the first hour of birth were evident. Continuous uninterrupted naked body contact from the moment of birth and for a full sixty minutes was experienced by twenty-six (33%) mothers and their newborn babies. Five (6.4%) mothers and their newborns were undisturbed for 43–56 minutes from the moment of birth. Only mothers who gave birth vaginally held their newborns in naked body contact within the first minute of birth (n = 43; 55%).

Naked body contact was interrupted within the first 5 minutes of birth for twenty-two (28%) mothers and their newborns. Eleven (14%) infants began contact immediately but were moved away from their mothers for resuscitation procedures or held by another person and then put back to maintain naked body contact for 37–58 minutes. Eleven (14%) began contact between two and five minutes then remained undisturbed for 34–59 minutes.

### Initial separation

‘Skin contact later’ was experienced by seventeen (21%) mothers and their newborn babies. One (1.2%) infant who commenced naked body contact immediately after birth but was interrupted during the first minute and not recommenced was categorised with infants who had no naked body contact. Seven (9%) mothers and their infants had no time in naked body contact during the first hour of birth. All mothers who had a caesarean section (n = 17; 22%) were initially separated from their babies for at least 24 minutes.

The most common event to separate newborns from their mothers during the first hour after birth was resuscitation intervention (n = 39; 50%) (facial oxygen, oronasopharangeal or gastric suction), followed by baby held by other person (n = 38; 48.7%), warming (n = 34; 43.6%), routine practices such as wrapping babies, vitamin K injection or weighing (n = 23; 29.5%), assessment of babies (n = 23; 29.5%), and mothers’ request to stop holding or not to hold her baby (n = 13; 16.7%). Resuscitation procedures were carried out on all babies born by caesarean section and on two thirds of babies born vaginally (n = 42 out of 61).

### Maternal behaviours

Three components of maternal behaviour to initiate breastfeeding identified by PCA were ‘state of nipple pain or trauma’ ‘getting ready’ and ‘position to entice and attach’. Fifty-nine mothers (75.6%) actively positioned themselves (n = 22; 28.2%) or their babies (37; 47.4%) to begin breastfeeding. Thirty-seven (47%) held their nipple to their babies’ mouth and five (6.4%) expressed colostrum to entice their baby to latch.

### Midwifery support of breastfeeding

Mothers were mostly offered help to breastfeed. Mothers received a variety of assistance. PCA results confirmed the definition for ‘assist to attach’ chosen for the independent variable.

Five descriptions for practices observed assisting mothers initiate breastfeeding emerged. Firstly, nine mothers (11.5%) received no assistance at all and only one of these was offered assistance. Secondly, seven mothers (9%) received ‘hands off’ midwifery support, meaning any verbal or nonverbal supportive instruction, encouragement or information without any ‘hands on assistance’. Most mothers (n = 62; 80%) received ‘hands on’ help. Three categories of ‘hands on’ help identified were, 1) ‘minimal assistance’ when helpers touched babies to position them optimally or closer to the breast; 2) ‘moderate assistance’ where helpers touched mothers’ breasts or babies to help babies attach to their mothers’ breast and 3) ‘full assistance’ help to both position and attach babies to mothers’ breasts. See Table 3 for descriptions of breastfeeding assistance mothers received.

**Table 3:** Descriptions of assistance mothers received to initiate breastfeeding in the birthing room by birth type

**Description of assistance**	**Birth type**		
	**Caesar**	**Vaginal**	**TOTAL**
	**n (%)**	**n (%)**	**n (%)**
**No assistance**	5 (6.4)	9 (11.5)	**9 (11.5)**
^**a**^**Hands ‘off’ support** (without other support)	0 (0)	7 (9)	**7 (9)**
Readiness of baby to breastfeed discussed			
Encouragement given			
Verbal instruction given			
Mother assisted to another position			
Mother’s hands guided to support baby			
Baby feeding behaviour noted			
Baby ability to breastfed affirmed			
Assisted to sit up – bed wound up			
**Hands ‘on’ position - minimal assistance**	3 (3.8)	22 (28.2)	**25 (32.1)**
Baby turned to face mother’s breast			
Mother reassured and praise given			
Instruction regards expressing			
Father moves baby over to nipple			
Baby positioned close to breast			
^**b,c**^**Hands ‘on’ attach - moderate assistance**	6 (7.7)	14 (17.9)	**20 (25.6)**
Baby’s neck held by tong grip^b^			
Mother’s breast shaped to help baby attach^b^			
Baby held to breast for mother^b^			
Back of baby head held^c^	3 (3.8)	14 (17.9)	**17 (21.8)**
Colostrum expressed for mother			
Breastfeeding assistance offered			

^a^Mothers who received hands ‘on’ help also received hands ‘off’ support.

^b^‘Hands on assistance to attach’ if any one of these.

^c^Back of baby head held with help to attach Independent variable (IV).

Full assistance – is assisted with both positioning and attachment.

The back of babies’ heads were held in 22% of total cases, sixteen of moderate assistance and one of minimal assistance. Fourteen (82%) of these mothers had given birth vaginally, ten (59%) of which experienced continuous uninterrupted naked body contact with their infant (34–60 minutes) and four (23%) interrupted naked body contact (44–59 minutes). Twelve out of fourteen infants achieved suckling, but only four (33%) suckled effectively. One infant sustained suckling for 25 minutes and then was held by the father and others in the room. One infant suckled for 23 minutes after several repeated attempts to grasp the nipple. Two babies suckled effectively for 4 minutes, one toward the end of the hour and the other was unable to reattach when the back of his head was held to assist. The eight infants who did not suckle effectively, took a few suckles over a 2–3 minute period with repeated attempts to attach and suckle. One baby failed to sustain suckling when his mother held the back of his head and was then placed lying on his back so he could not reach the nipple for further attempts.

All three (3.8%) of the infants born by caesarean section commenced suckling within the hour. However, the one who had no maternal-infant naked body contact suckled effectively. Of the two in naked body contact with their mothers, one suckled effectively for seven minutes, the other suckled off and on for seven minutes and the mother reported nipple pain toward the end of the hour.

### Chi-square analyses

Chi-square test for independence (with Yates continuity Correction) indicated ‘suction’, ‘chin contacts breast’ and ‘parity’ had significant associations with ‘effective suckling’ (all p-values < 0.05) (see Table 4).

**Table 4:** Chi-square analyses of associations between ‘effective suckling’ and proposed predictor variables

**Independent variables**	**Proportion effective suckle n (%)**	**Χ**^**2**^	***p***
**Parity**			
Primipara	7 (9)	8.778	.003
Multipara	23 (29.5)		
**Birth type**			
Caesarean section	4 (5.1)	1.321	.250
Vaginal	26 (33.3)		
**Analgesia**			
No	18 (23.1)	.000	1.000
Yes	12 (15.4)		
**Suction**			
No	28 (35.9)	8.563	.003
Yes	2 (2.6)		
**Baby’s chin nudges mother’s breast**			
No	4 (5.1)	6.508	0.011
Yes	26 (33.3)		
**Back of baby head held & assist to attach**			
No	25 (32.1)	.343	.558
Yes	5 (6.4)		
**Skin-to-skin contact start & duration**			
Continuous uninterrupted skin-to-skin contact	11 (14.1)	3.685	0.298
Interrupted skin-to-skin contact	11 (14.1)		
Skin-to-skin later	7 (9)		
No skin-to-skin contact	1 (1.3)		

### Logistic regression

A direct logistic regression analysis was performed to assess the impact of four factors on the likelihood of newborn infants suckling effectively during the first hour of life. Predictor variables entered into the model were parity, suction, chin contacts breast, and duration of naked body contact. The hypothesis that infants placed naked on their mother’s bare chest from the moment of birth and left uninterrupted for at least 30 minutes after birth would breastfeed effectively was not confirmed (*p* = .142).

To assess the impact of assistance mothers received on effective suckling, the model was rerun with predictor variables parity, suction, chin contacts breast and baby head held with assistance to attach. Although infants were somewhat less likely to suckle effectively if the back of their head was held when mothers received assistance to attach their newborn to the breast, the 95% CI for the odds ratio included 1, indicating no real difference.

The final model suggested thirty-five percent of the variability in effective suckling is explained by three variables. Newborns were almost six times less likely to suckle effectively if naso-oropharyngeal suction was administered at birth (OR .176; CI .04 - .9). Babies were four times more likely to suckle effectively when their chin made contact with their mother’s breast as they approached the nipple (OR 3.8; CI 1.03 - 14). Likewise, infants of multipara mothers were four times more likely to suckle effectively (OR 4; CI 1.32 - 12) than first time mothers. Results of the direct logistic regression analysis model are presented in Table 5.

**Table 5:** Logistic regression predicting likelihood of ‘effective suckling within the first 60 minutes of life

					**95% CI, Odds Ratio**	
**Predictor**	**β (SE)**	**Wald**	***p***	**OR**	**Lower**	**Upper**
Suction (n = 21)	-1.735 (.830)	4.371	.037	.176	.035	.897
Chin nudges breast (n = 53)	1.331 (.665)	4.007	.045	3.784	1.028	13.927
Parity – Multipara (n = 42)	1.383 (.566)	5.969	.015	3.988	1.315	12.097
Constant	-1.930 (.719)	7.194	.007	.145		

Nagelkerke R^2^ = .35.

Hosmer and Lemeshow Chi-square, .695, df = 5, p = .983.

Percent correctly classified 74.4%.

## Discussion

The key findings of this study are that effective suckling within the first hour of birth can be predicted by a model of best practice, including positioning babies so they can instinctively nudge their chin into the underside of their mother’s breast as they approach the nipple and attach to suckle.

This study has several unique features. First, the study reported detailed observations of what actually happened in the birthing room for the first hour. The use of video recording could miss some activities. Second, the detailed observations of infant feeding behaviour and maternal assistance received provide definitions for both suckling commencing and assistance. Third, the analysis identified predictors of effective suckling in the first hour of life that have not been reported elsewhere.

The measure used as the outcome variable for effective suckling was that infants grasped the nipple, commenced and sustained suckling for more than three minutes. Previous researchers have described both ‘reaching the nipple’ and ‘effective suckling’ as outcome variables but the latter has been criticised as subjective [20,62]. Researchers who measured ‘effective suckling’ as an outcome may not have used reliable tools and their definitions need more clarity for observer agreement [62,63]. Published tools designed to observe effective breastfeeding have also been criticised as subjective [64,65]. Observations in this study concur with descriptors commonly documented by midwives and provide clear definitions of effective suckling for clinical application and future research. Further insight into effective suckling at the first breastfeed is needed by analysis of time infants sustained suckling.

Naked body contact was interrupted on many occasions for both newborns placed immediately on their mothers’ bare chest and those placed in naked body contact after initial separation. The wide range of inconsistent practices observed may be explained in part by maternal choice at the time. For example, some mothers gave birth in positions where it was not conducive or safe to hold their naked baby immediately (such as standing up leaning over the bed). Others started holding their naked baby but requested another person to hold the baby while they got comfortable or needed brief medical attention. In several cases, mothers wanted to take time to look at and examine their baby and adapt to the fact of having just given birth before holding their naked newborn on their bare chest. Results of this study validate revised BFHI Global criteria accreditation guidelines in Australia that newborns are placed “with their mothers in skin-to-skin contact immediately *or within five minutes* after birth” (p.11) [27].

The frequent interruptions of naked body contact observed in this study may have interfered with some infants achieving suckling and suckling effectively within the hour. Righard and Alade reported that on average newborns begin active feeding behaviours at around twenty minutes and this is a crucial time for infants to not be disturbed [20]. On the other hand not all babies are ready to begin feeding so soon after birth. Another factor known to interfere with effective suckling in the newborn is analgesic drugs [58]. Twenty out of forty- eight infants who did not suckle effectively may have been adversely affected by analgesia. However, analgesia such as provided by pethidine or epidural administration did not show as a negative predictor of suckling in the model tested for this study.

Given that over half of the babies were placed immediately with their mothers but naked body contact was disturbed for a third of them (and multiple times for several cases), it was not possible to determine the importance of continuous uninterrupted naked body contact as a predictor of effective suckling within the hour. Since the study ended after 60 minutes it is not known how many newborns suckled effectively over the next few hours or if they remained in naked body contact. Widstrom et al. [15] emphasises the importance of undisturbed naked body contact to enable newborns to find the nipple using all their senses and commence suckling. This growing evidence warrants revision of BFHI global criteria and accreditation standards to ‘Step 4’ [2]. Such revisions could contribute to continuity of naked body contact instead of multiple interruptions observed in the present study.

### Position of “chin on breast”

Findings revealed some important insights about positioning and attachment at the breast. When the baby’s chin is in contact with the breast, the gape reflex is elicited enabling the baby to gape widely and take an adequate amount of breast tissue as observed by Widstrom et al. [15,18]. The odds of infants achieving suckling within the hour were significantly increased when their chin pressed into their mother’s breast when approaching the nipple. This supports a possible link between naked body contact and ‘instinctive positioning’ for optimal latch as recommended by experienced midwives and lactation consultants [40,66-69]. The four categories of primitive neonatal reflexes observed by Colson et al. [70] (endogenous, motor, antigravity and rhythmic) and nine behavioural phases described by Widstom et al. [15] (birth cry, relaxation, awakening, activity, crawling, resting, familiarization, suckling and sleeping) along with observations from the present study could be used as a guide for midwives and help mothers interpret newborn feeding behaviours for readiness to feed.

Observation and working with newborn feeding behaviour is supported by revised BFHI guidelines [2,27]. However traditional education materials (pictures/diagrams) explicitly instruct midwives to teach mothers to elicit the gape reflex by holding the baby away from their body then ‘quickly bring the baby to the breast’ [27,45,71-73]. This instruction may be a source of confusion to mothers, when *‘correct’* positioning and *‘correct’* attachment are emphasised in the postnatal period [7,42,74,75]. Despite revised global criteria teaching materials there remains disparity between teaching positioning and attachment in the birthing room and for subsequent breastfeeds. Further research with large numbers of birthing women could inform future revisions of BFHI criteria. Perhaps a number of BFHI accredited facilities could pool data and examine these issues more closely.

### Assistance mothers received

Descriptions of assistance mothers receive are useful measures for clinical application, education and further research. Results of this study quantify terms commonly used in midwifery practice to describe assistance mothers receive to initiate breastfeeding. To our knowledge no formal definitions of these terms have been published to date.

Although breastfeeding is perceived to be a learnt skill with mothers requiring help to position and attach baby *‘correctly’,* many of the women actively positioned themselves or their babies to begin breastfeeding without any instruction. To date, BFHI guidelines have not adequately defined newborn readiness to begin feeding or the nature of help to give, yet stipulate that help be given within 30 minutes [2]. Even though it is advantageous for babies to feed within an hour of birth, [26] it may be harmful to coerce them before they are ready [18,56]. Descriptions evident from observing assistance mothers received provide guidance for teaching midwives how to help mothers.

Amongst mothers and infants who received ‘assistance to attach’, the back of the baby’s head was held for almost half the cases (46%). The greater proportions of these were mothers who gave birth vaginally and who held their babies in naked body contact continuously from the start or with only a short interruption at the beginning. This may explain lack of support for the research hypothesis. Holding the back of the baby’s head at a feeding attempt can be counterproductive and lead to breast refusal [59]. Results suggest a number of factors contribute to effective suckling in the first hour of life other than duration of maternal-infant naked body contact. Results do suggest the possibility of infants being less likely to suckle effectively if the back of their head was held to assist attachment. This phenomenon needs further investigation. Research to date reporting positive effects of mothers being assisted with positioning and attachment have been conducted during the postnatal period after the first breastfeeding attempt [75-78]. Further analysis is needed to determine associations between effective suckling in the birthing room, type of assistance mothers’ receive and postnatal breastfeeding problems such as nipple trauma and breast refusal.

Further research is warranted to test differences between birth types. BFHI Global Criteria for ‘Step 4’ advocates that mothers who have a caesarean section without general anaesthesia be offered the same care as mothers who give birth vaginally [2]. While barriers to immediate skin-to-skin contact for mothers having a caesarean section have been reported in the research literature, all mother infant pairs had the same chance of meeting the recommended 60 minutes [79,80]. The hospital was preparing for BFHI accreditation and many staff had completed education outlining best practice. Further research is needed to explore whether mothers are informed about the importance of naked maternal-infant body contact in helping newborn feeding ability and coordination to attach to the breast independently.

Observations ceased at 60 minutes so it is not possible to know how many continued on in maternal-infant naked body contact and fed well before being disturbed within a 2–3 hour timeframe. It is known that there should not be a rush to have the baby feed in the first hour [15]. It is possible that less “hands on help” may be required if more emphasis were placed on the importance of skin-to-skin contact to coordinate feeding behaviours. Future revisions to BFHI and policy could be informed by further research.

### Oro-nasopharyngeal suction interventions

The adverse effect of suction on newborn feeding ability needs attention in midwifery education programs. In this study, the likelihood of babies achieving suckling within the hour diminished if suctioning intervention was administered and confirms available evidence [17,20,21]. Routine suctioning of newborns is opposed by The American Academy of Pediatrics. The small percentage of babies that may need suction at birth can be assessed initially while on their mother’s chest [59,81,82]. Considering the known benefits of naked body contact between mother and newborn for thermoregulation, stabilisation of heart and respiratory rates, reduced stress and crying there seems little justification for routine suction [9,83].

Results of the present study need to be considered in light of limitations concerned with sample size, “Hawthorne effect” and practice changes since data collection. Although recruitment allowed for losses, there were more missed births than anticipated, reducing the overall sample size. However, in comparison to small samples (n = 21) in other observational studies e.g. Widstrom et al., [17] the present sample of 78 was considered adequate. It could be that midwives’ practices altered as a result of being observed and do not truly reflect actual practice (Hawthorne effect) [50]. However, given that best practice guidelines were not adhered to in every case, it is possible that participating midwives were performing in their usual manner. Although data were collected in 2005 and practice development may have addressed some of the deficiencies identified, the results of this study continue to be pertinent.

## Conclusion

Suckling commencement within the first hour after birth is associated with a collective model of practices including infants held and positioned so their chin can instinctively nudge the underside of their mother’s breast as they approach to grasp the nipple and attach to suckle. It is the type of help given in the birthing room that midwives need to pay attention to. The help provided is best if done in conjunction with, paying attention to newborn feeding behaviour s and readiness of babies to begin suckling, no unnecessary suction and if any hands on assistance to attach is needed it should be gentle with helping babies’ chins to approach the breast first.

## Abbreviations

PCA: Principal Components Analysis; DV: Dependent variable; IV: Independent variable; IVs: Independent variables; BFHI: Baby Friendly Hospital Initiative; IBCLC: International Board Certified Lactation Consultant; SPSS: Statistical Package for the Social Sciences; IBM: International Business Machines.

## Competing interest

The authors declare that they have no competing interests.

There are no financial or non-financial competing interests (political, personal, religious, ideological, academic, intellectual, commercial or any other) to declare in relation to this manuscript.

## Authors' contributions

RC was involved in the conception and design of the study, protocol writing, designing and analysis of the observation tool, data collection, analysis and interpretation of the results, and in drafting the manuscript and was the lead author on the manuscript. DC was involved in the conception and design of the study, interpretation of the results, and in drafting the manuscript and critical review of the manuscript for significant intellectual input. MC assisted in conception and study design, and critical review of the manuscript. FD assisted in conception and study design and critically reviewed the manuscript for substantial intellectual input. All authors read and approved the final manuscript.

## Authors’ information

RC is a registered nurse, midwife and International Board Certified Lactation Consultant at Metro South Hospital and Health Service, Queensland Health and was the researcher undertaking this observation study (January to July 2004). DC is Professor at Griffith Heath Institute. MC is Professor of School of Nursing and Midwifery and Griffith Heath Institute. FD is Professor of Maternal and Infant Health and director of MAINN.

## Pre-publication history

The pre-publication history for this paper can be accessed here:

http://www.biomedcentral.com/1471-2393/14/20/prepub
